# Facile Preparation of High-Performance Reduced Graphene Oxide (RGO)/Copper (Cu) Composites Based on Pyrolysis of Copper Formate

**DOI:** 10.3390/ma17112519

**Published:** 2024-05-23

**Authors:** Zhendong Shi, Qingwen Yun, Tong Zhang, Changsheng Xing, Jie Li, Yunzhong Wu, Lidong Wang

**Affiliations:** 1 Harbin Aircraft Industry Group Co., Ltd., Aviation Industry Corporation of China, Harbin 150066, China; yqw_1986@163.com; 2 Harbin Hafei Aviation Industry Co., Ltd., Aviation Industry Corporation of China, Harbin 150010, China; 3 School of Materials Science and Engineering, Harbin Institute of Technology, Harbin 150001, China; 13796010932@163.com (T.Z.); xingcs7@163.com (C.X.); 19b909024@stu.hit.edu.cn (J.L.); 20b909010@stu.hit.edu.cn (Y.W.)

**Keywords:** carbon materials, metal composites, mechanical property, electrical conductivity, thermal conductivity

## Abstract

Graphene has attracted much interest in many scientific fields because of its high specific surface area, Young’s modulus, fracture strength, carrier mobility and thermal conductivity. In particular, the graphene oxide (GO) prepared by chemical exfoliation of graphite has achieved low-cost and large-scale production and is one of the most promising for Cu matrix composites. Here, we prepared a high strength, high electrical conductivity and high thermal conductivity reduced graphene oxide (RGO)/Cu composite by directly heating the GO/copper formate. The oxygen-containing functional groups and defects of RGO are significantly reduced compared with those of GO. The tensile yield strength and thermal conductivity of RGO/Cu composite with RGO volume fraction of 0.49 vol.% are as high as 553 MPa and 364 W/(m·K) at room temperature, respectively. The theoretical value of the tensile yield strength of the composite is calculated according to the strengthening mechanism, and the result shows that it agrees with the experimental value. After hot-rolling treatment, the ductility and conductivity of the composite materials have been greatly improved, and the ductility of the RGO/Cu composite with RGO volume fraction of 0.49 vol.% has been increased to four times the original. This work provides a highly efficient way to fabricate a high-performance RGO-reinforced Cu composite for commercial application.

## 1. Introduction

Two-dimensional materials are widely used in bipolar transistors, non-volatile memory devices, functional metamaterials and other applications [1,2,3,4]. Graphene has aroused great research interest in many scientific areas because of its specific surface area of ~2630 m^2^g^−1^ [5], Young’s modulus of ~1100 GPa [6], fracture strength of 125 GPa [6], carrier mobility of 200,000 cm^2^ V^−1^s^−1^ [7] and thermal conductivity of ~5000 Wm^−1^K^−1^ [8] in recent years. Scientists have developed many general and reliable synthetic routes for the preparation of graphene and its derivatives to further utilize these properties in a variety of applications. It ranges from the top-down exfoliation of graphite to the bottom-up epitaxial growth. Particularly, the chemically exfoliated graphene oxide (GO) sheets have achieved low-cost and mass production. The GO sheets have many active oxygen-containing groups and can be used for further functionalization and adjustment of performance [9,10,11,12].

In recent years, Cu materials with excellent electrical and thermal conductivity have been used in fields such as aerospace, automotive, electronics, etc. As a reinforcing material for copper, graphene can effectively improve the mechanical and physical properties of copper. So far, scientists have proposed four major mechanisms for the improvement of yield strength, namely the thermal expansion mismatch causes the geometrically necessary dislocation (ΔσCET), grain refinement (ΔσHall−Petch), load transfer from Cu matrix to RGO (ΔσLT) and Orowan strengthening (ΔσOrowan) [13,14]. Graphene can effectively enhance the electrical performance of materials when combined with copper. This is attributed to the injection of a high concentration of electrons from copper into graphene, which provides a more efficient pathway for their transmission. Additionally, graphene itself has a high thermal conductivity, so combining it with copper can improve the thermal properties of the material, which is also confirmed in previous reports [15,16]. The graphene/copper composite material finds extensive application in various fields, including conductive lead frames, specialized wires, and heat dissipation materials, owing to its exceptional mechanical strength, corrosion resistance and distinctive physical properties that cater to the demands of copper materials in extreme environments [17,18,19,20,21].

Many methods have been developed to prepare reduced graphene oxide (RGO)-reinforced Cu matrix composites using GO as raw material, such as molecular-level mixing (MLM), the impregnation process and ball milling, the electrodeposition method, the in situ growth method, etc., and the strengths of composites are generally improved. Hwang et al. [17] used MLM to disperse GO sheets into a Cu matrix and the tensile yield strength of the composite materials reached 284 MPa. Yang et al. [18] prepared a hierarchical layered RGO/Cu composite by adjusting the temperature and pH value of solutions during the MLM process, and the tensile strength of the composite was as high as 748 MPa. Xiong et al. [19] successfully fabricated nacre-like RGO/Cu composites by a procedure for impregnation, and then the tensile yield strength of the composite only was 233 MPa. However, the MLM and impregnation processes are complicated and difficult for industrial application. Yu et al. [20] fabricated RGO/Cu composites via electrodeposition, exhibiting a yield strength of 277 MPa and an electrical conductivity of 91.3% IACS. Xing et al. [21] employed the in situ growth method to prepare oriented bimodal Gr/Cu composite material, achieving a yield strength of 353 MPa and an electrical conductivity of 91.9% IACS. However, the prolonged electrodeposition process and high-temperature carbonization procedure contribute to increased energy consumption during production. Compared to the previous method, the ball milling technique offers simplicity and low energy consumption, enabling uniform mixing of graphene and copper. According to Yue [22] et al., a tensile strength of 230 MPa was achieved by fabricating graphene nanosheets/Cu composites using ball–milling GO and Cu powder. However, the strength of the composite is generally mediocre. Therefore, there is a need for an efficient preparation method to fabricate high-performance RGO/Cu composites suitable for commercial applications.

It has been reported that an appropriate increase in the volume fraction and quality of graphene can improve the properties of composites [23,24]. Our previous research involved the fabrication of carbon nanosheet-reinforced Cu matrix composites directly using pyrolyzing copper formate [25]. However, the composites were not as strong as expected due to a low volume fraction and quality of carbon.

To efficiently and energy-savingly prepare high-performance RGO/Cu composite materials, a high-efficiency method is proposed to fabricate high-performance RGO/Cu composites based on pyrolysis of copper formate. We are directly heating the GO/copper formate into RGO/Cu composite powder at 400 °C in H_2_/Ar mixed gas, and the RGO/Cu composite is obtained through spark plasma sintering (SPS). The functional groups that contain oxygen in RGO are significantly less and have fewer defects than those in GO. The RGO/Cu composites are characterized by their excellent mechanical, electrical and thermal properties.

## 2. Experimental

### 2.1. The Preparation of the RGO/Cu Composite Powders

GO aqueous solution (18.5 mg/g) was provided by Hangzhou GaoXi Technologies Co. (Hangzhou, China) and Wengjiang Reagent gave us copper formate tetrahydrate (Wengjiang, China). A mixture of GO and 142 g of copper(II) formate tetrahydrate was blended with 180 mL of ethanol solution, then placed in a 1.5 L stainless steel jar and ground at 300 rpm for 12 h. The grinding media consisted of three types of balls with diameters of 10 mm, 2 mm and 1 mm in a ratio of 1:1:1. Additionally, the mass ratio between the grinding ball and the mixture of copper formate/RGO was maintained at 3:1. After grinding stainless steel balls and copper formate tetrahydrate together, the samples were dried in a vacuum oven at 120 °C for 4 h, respectively. In this process, copper formate did not undergo decomposition [25]. After obtaining the powders, they were heated at 400 °C (10 °C/min) for 3 h in a tube furnace with an H_2_ content of 17% until they were derived as the RGO/Cu composite powders.

### 2.2. The SPS and Hot Rolling of the RGO/Cu Composites

RGO/Cu composite powders were pressed in a graphite mold using SPS-1050 under an external pressure of 40 MPa for 5 min at 700 °C to produce the composite materials and the SPS-1050 was produced by the Sumitomo carboniferous company, Tokyo, Japan. The RGO/Cu composites that were sintered had a diameter of 30 mm and a thickness of 5 mm. The hot-rolling process at 500 °C reduced the RGO/Cu composites by 70%, and they became RGO/Cu-R composites, where R presents the hot-rolling process.

### 2.3. The Characterization of the RGO/Cu Composite Powders and Composites

The Ben Yuan CSPM 5600 scanning probe microscope was used in tapping mode to obtain an image of atomic force microscopy (AFM). The Helios Nanolab 600i has an energy-dispersive X-ray detector (EDX) that can be used for scanning electron microscopes (SEM). A high-frequency infrared carbon sulfur analyzer (CS-901B, Beijing Wanlian Daxinke Instruments) was utilized to measure the weight percentage of carbon in composites (Beijing, China). The composites were analyzed using the Raman spectrum, a 532 nm laser from the B&W Tek confocal micro-Raman spectrometer. The use of Fourier transform infrared spectroscopy (FTIR, Bruker Vertex 70, Mannheim, Germany) was employed to examine the surface chemistry and structural characteristics of GO and RGO powder. Using Cu Kα radiation (λ = 1.54 Å), X-ray diffraction (XRD, a Philips X’Pert X-ray diffractometer, Amsterdam, The Netherlands) was employed to analyze the phase of the composite. Analysis using X-ray photoelectron spectroscopy (XPS, Thermo Fisher spectrometer, Waltham, MA, USA) revealed the bonding characteristics of GO and RGO. The calibration of all peaks of the XPS spectra was carried out based on the peak position of C 1s (284.6 eV). To characterize the microstructures of the composite powders and composites, an energy-dispersive X-ray detector was used by using a transmission electron microscope (TEM, Talos F200x, Waltham, MA, USA). Electron Backscattered Diffraction (EBSD) was used to analyze the morphology of the specimen’s grain.

The Instron-5569 electronic universal testing machine was used to perform room-temperature tensile tests on RGO/Cu composites at a crosshead speed of 0.5 mm/min. A wire electrical discharge machine was used to cut the tensile specimens, which were 15 mm long, 2 mm wide and 1 mm thick, respectively. A CTA-3 instrument from Beijing Cryoall Science and Technology Co., Ltd. (Beijing, China) was utilized to measure the electrical conductivities using the four-probe method. The wire was used to cut the specimens to dimensions of 2 mm × 2 mm × 18 mm. To determine the specific heat (Cp) of composites, a differential scanning calorimeter (DSC; PerkinElmer DSC 8000, Branson, MO, USA). The thermal diffusivity (α) of the composites was measured by a laser thermal conductance instrument (LFA457, Germany) in the temperature range of 25 to 400 °C.

## 3. Results and Discussion

### 3.1. The Fabrication Process of RGO/Cu Composite

**Figure 1:**
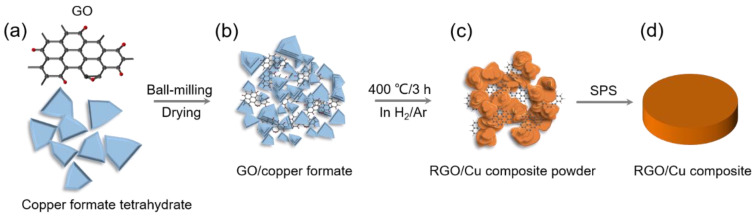
A diagram of the fabrication process for RGO/Cu composite. (**a**) Raw materials: GO and copper formate tetrahydrate. (**b**) The GO/copper formate composite powder obtained by ball milling and drying. (**c**) The obtained RGO/Cu composite powder by heat treatment in H_2_/Ar atmosphere. (**d**) Sintered RGO/Cu composite through spark plasma sintering.

The fabrication process for the RGO/Cu composite is depicted schematically in Figure 1. Firstly, the mixture of GO and copper formate tetrahydrate was thoroughly blended with ethyl alcohol using ball milling. The sample was placed in a vacuum at 120 °C for four hours. Secondly, the mixtures of GO and copper formate were heated to 400 °C for 3 h in H_2_/Ar atmosphere and then cooled to room temperature with the furnace. Finally, the RGO/Cu composite powders that were obtained underwent sintering and densification through SPS.

### 3.2. Microstructural Characterizations of GO and RGO

The weight percentages of carbon were used to accurately calculate the volume fractions of carbon in composites, as shown in Table 1. Carbon and Cu densities of 2.2 and 8.96 g/cm^3^ are assumed to be in this calculation. It is assumed that the carbon remaining from the decomposition of copper formate in each composite is the same as the carbon volume fraction (0.1 vol.%) of the RGO/Cu(0) material without GO added. Thus, the volume fraction of RGO in a composite is the volume fraction of carbon in each composite minus 0.1 vol.%. To simplify things, RGO/Cu (α) is used to abbreviate the composites, with (α) vol.% being the volume fraction of RGO (0, 0.19, 0.49, 0.64, 0.85 or 13.1) vol.%, respectively.

**Figure 2:**
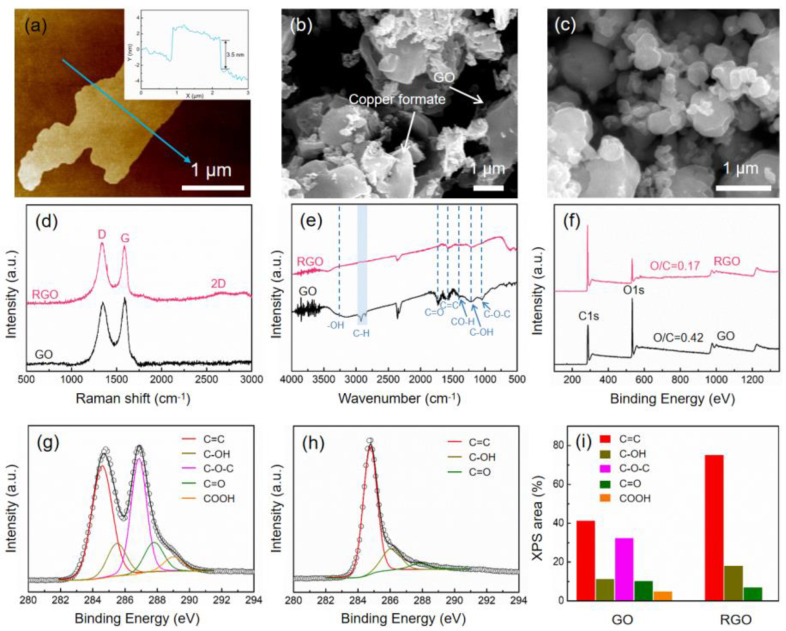
(**a**) AFM image and the thickness of GO. SEM images of (**b**) GO/copper formate and (**c**) RGO/Cu composite powders. (**d**) Raman spectra, (**e**) FTIR spectra and (**f**) XPS survey spectra of GO and RGO. C 1s spectra of (**g**) GO and (**h**) RGO utilized by employing the Gaussian-Lorentzian peak shape model. (**i**) The XPS area in the C 1s peak is used to estimate the contents of oxygen functional groups.

The thickness of GO flakes was measured by using AFM. An individual GO flake with a thickness of ~3.5 nm can be found in Figure 2a, which is the typical four-layer thickness of GO [26]. The SEM image of GO (Figure S1) shows a transparent and gossamer structure. Figure 2b shows the SEM image of the mixture of GO/copper formate, which indicates that GOs were homogeneously distributed in the copper formate without agglomeration. The morphology of RGO/Cu(0.49) composite powder is shown in Figure 2c. The RGO was not obvious to observe, indicating that the RGOs were uniformly dispersed between submicron and nanometer copper particles. The other RGO/Cu composite powders have SEM images displayed in Figure S2. It has been observed that the copper particle size first decreases and then increases as the volume fraction of RGO increases. The typical D-bands of ~1350 cm^−1^ and ~1600 cm^−1^ are displayed by the Raman spectra (Figure 2d). Functionalization-induced amorphization causes a drastic suppression of the 2D peak at ~2700 cm^−1^, which is attributed to the six-membered ring’s out-of-plane vibration mode [27]. It shows GO is reduced to RGO [28]. The surface functionalities of the GO and RGO were examined by FTIR spectroscopy. Figure 2e shows several distinct absorption bands of oxygen-containing functional groups in GO, such as O–H (3000–3600 cm^−1^), C-H (~2921 and ~2856 cm^−1^), C=O (~1715 cm^−1^), C=C (1500–1600 cm^−1^), C–O–H (~1401 cm^−1^), C–OH (~1217 cm^−1^) and C–O–C (~1048 cm^−1^) [29]. However, the intensity of these groups decreased significantly or almost disappeared in RGO. The surface composition and chemical configuration of GO and RGO are analyzed quantitatively using X-ray photoelectron spectroscopy (XPS). As shown in Figure 2f, XPS of GO and RGO exhibit the peaks of C 1s (284.6 eV) and O 1s (531 eV). It can be found that the O/C atomic ratio of RGO (0.17) is lower than that of GO (0.42). The Gaussian–Lorentzian peak shape model was used to fit the C 1s spectra of GO and RGO, which are depicted in Figure 2g,h, respectively. At ~284.6 eV, ~285.5 eV, ~286.6 eV, ~287.6 eV and 288.8 eV, the components of C=C, C–OH, C–O–C, C=O and COOH can be found. According to the fitted C 1s peck area, Figure 2i illustrates the calculation of the relative contents of oxygen functional groups. The contents of C=C and C–OH increase and the relative contents of C–O–C, C=O and COOH decrease or almost disappear in RGO as compared to those in GO. Based on the evidence from Raman, FTIR and XPS experiments, we can conclude that after GO is reduced to RGO, the number of oxygen functional groups was reduced or even disappeared and defects significantly decreased.

### 3.3. Microstructure Characterizations of the Composites

**Figure 3:**
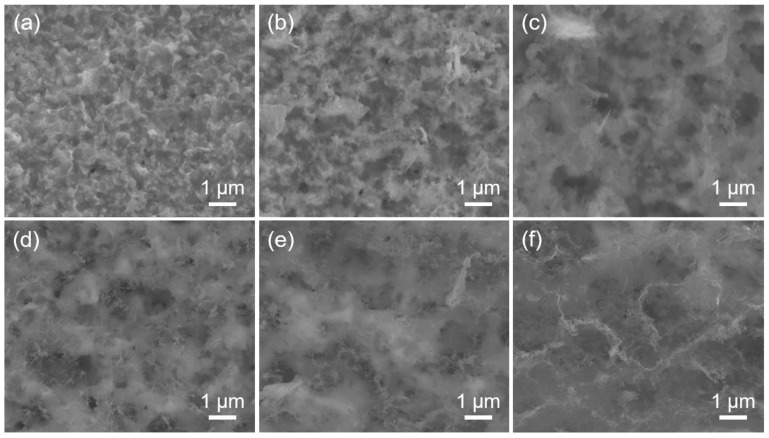
SEM images of the surfaces diluted 10 wt. % nitric acid and allow it to sit for 90 min (**a**) RGO/Cu(0), (**b**) RGO/Cu(0.19), (**c**) RGO/Cu(0.49), (**d**) RGO/Cu(0.64), (**e**) RGO/Cu(0.85) and (**f**) RGO/Cu(1.31).

Five peaks in the XRD patterns of the six RGO/Cu composites correspond to Cu (PDF No. 85-1326), whereas copper oxides do not have any peaks, as shown in Figure S3. SEM images (Figure 3) present characteristics of the surfaces of six composites after exposing them to dilute 10 wt. % nitric acid and allow it to sit for 90 min. Figure 3 demonstrates that the increase in RGO volume fraction increases the amount of RGO, and the etched RGO/Cu composites have a uniform distribution of RGO on the surface. In Figure 3f, the RGO on the surface of the RGO/Cu composite with etching accumulates to form a three-dimensional network structure as the volume fraction of the RGO increases.

**Figure 4:**
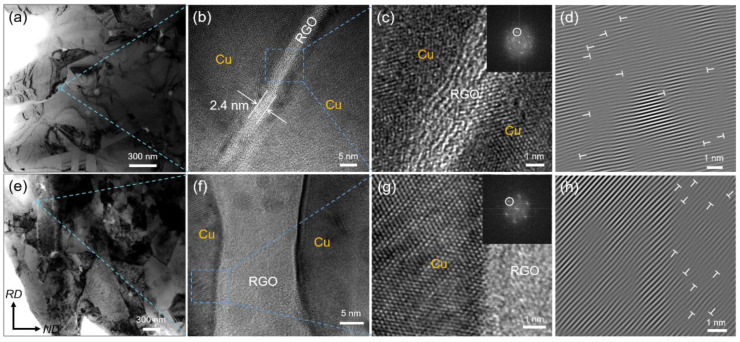
(**a**,**b**) TEM and (**c**) HRTEM images of RGO/Cu(0.49) composite, the corresponding FFT is shown in the upper right inset, (**d**) the corresponding inverse FFT figure of the inset in (**c**), (**e**,**f**) TEM and (**g**) HRTEM images of RGO/Cu(0.49)–R composite, the corresponding FFT is shown in the upper right inset, (**h**) the corresponding inverse FFT figure of the inset in (**g**).

Microstructures of RGO/Cu(0.49) and RGO/Cu(0.49)-R composites were characterized by TEM as shown in Figure 4. The surface of the RGO/Cu (0.49) composite is smooth without any noticeable holes or microcracks, as demonstrated in Figure 4a,b, and it can be seen that there is a RGO layer between the Cu matrix with a thickness of ~2.4 nm. A tightly interfacial structure in the composite has been revealed by the high-resolution TEM of RGO/Cu (0.49), which does not show any visible interfacial voids or cracks between RGO and the copper matrix, as shown in Figure 4c. The corresponding FFT and inverse FFT were shown in the upper right inset of Figure 4c and Figure 4d, respectively. A large distortion area can be clearly recognized as interface mismatch dislocations, which are indicated by “T” symbols. The high-density dislocation zone can be formed at the interface between the RGO and Cu matrix due to the different thermal expansion coefficients during the cooling of the SPS process. TEM photos of RGO/Cu (0.49)-R composite can be seen in Figure 4e,f. In the composite, the crystal grains in the rolling direction (RD) appear to be almost elongated. RGO has a tendency to be in line with the RD, which corresponds to the white stripes in the photo. The high-resolution TEM of RGO/Cu(0.49)-R is shown in Figure 4g. The interface between RGO and Cu exhibits no holes and microcracks, indicating the interface did not crack during the hot-rolling process. The corresponding FFT and inverse FFT were shown in the upper right inset of Figure 4g and Figure 4h, respectively. Similar to sintered composites, there are many dislocations at the interface between the RGO and Cu matrix in the RGO/Cu(0.49)-R composite. Lots of misfit dislocations at the RGO/Cu interface play an important role in strengthening composites due to stronger interaction between dislocations during the composite deformation process.

**Figure 5:**
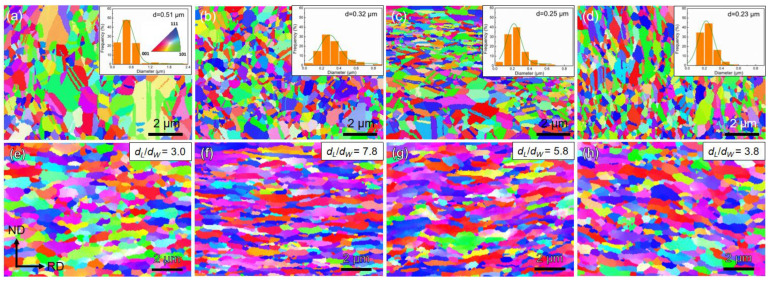
The EBSD characterization analysis, IPF maps and distribution of the grain size of the (**a**) RGO/Cu(0), (**b**) RGO/Cu(0.49), (**c**) RGO/Cu(0.85) and (**d**) RGO/Cu(1.31) composites. IPF maps of the (**e**) Cu matrix-R, (**f**) RGO/Cu(0.49)-R, (**g**) RGO/Cu(0.85)-R and (**h**) RGO/Cu(1.31)-R composites. The copper grains’ length and width directions are identified by *d_L_* and *d_W_*, respectively. The copper grains’ length and width directions are indicated by *d_L_* and *d_W_*, respectively, the insets show the average ratio values of *d_L_/d_W_* for the RGO/Cu-R composites.

Figure 5 shows that the EBSD characterization analysis helped us better understand their crystallography texture for RGO/Cu and RGO/Cu-R composites. The randomly distributed grain orientation can be seen in the inverse pole figure (IPF) maps (Figure 5a–d). The RGO/Cu(0), RGO/Cu(0.49), RGO/Cu(0.85) and RGO/Cu(1.31) composites have average grain sizes of 0.51, 0.32, 0.25 and 0.23 μm, respectively. The average grain sizes of the RGO/Cu composites decrease as the volume fraction of RGO increases, which is what this indicates. The grain orientation of the RGO/Cu composites in Figure 5a–d is randomly distributed as indicated by the IPF maps. The RGO/Cu(0), RGO/Cu (0.49), RGO/Cu (0.85) and RGO/Cu (1.31) composites have grains with average sizes of 0.51, 0.32, 0.25 and 0.23 μm, respectively. It can be seen that increasing the volume fraction of RGO results in a decrease in the average grain sizes of RGO/Cu composites. The IPF maps of RGO/Cu-R composites are shown in Figure 5e–h. The copper grains in the composites appear to have elongated along the RD. The copper grains’ length and width directions are referred to as *d_L_* and *d_W_*, respectively. The composites with greater deformation are indicated by the higher average *d_L_*/*d_W_* ratios. As shown in the inset of Figure 5e–h, the RGO/Cu-R composites have an average *d_L_*/*d_W_* ratio value of 3.0, 5.8, 7.8 and 3.8. As the volume fraction of RGO increases, so does the average *d*_L_/*d*_W_ ratio value, which increases initially but then decreases. The RGO is randomly distributed and rotates in line with the RD during the hot-rolling process, while the rolling process is bound to extend the copper grains along the RD, limiting their growth in the normal direction (ND). However, the decrease in RGO/Cu(1.31)-R composite could be ascribed to the fact that the excess RGO aggregates in the copper matrix and the deformation of copper grains could not be effectively prevented.

### 3.4. Mechanical Property and Strengthening Mechanisms of Composites

**Figure 6:**
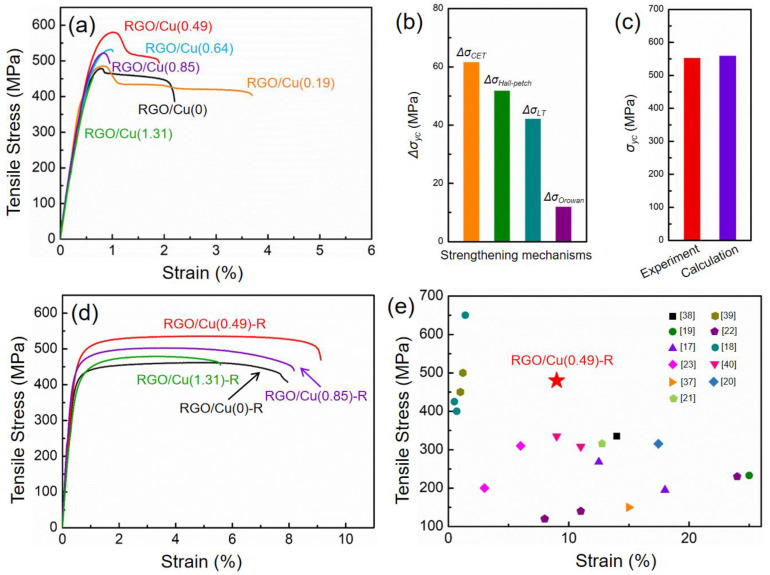
(**a**) Tensile and stress–strain curves of RGO/Cu composites, (**b**) theoretical calculation of different strengthening mechanisms in the RGO/Cu(0.49) composite, (**c**) the comparison between the experimental and theoretical calculation, (**d**) tensile and stress–strain curves of the RGO/Cu-R composites, (**e**) tensile yield strengths versus the strain of RGO/Cu composites in comparison with available data from the literature [17,18,19,20,21,22,23,37,38,39,40].

The representative true tensile stress–strain curves of RGO/Cu composites shows that the tensile yield strengths of RGO/Cu(0), RGO/Cu(0.19), RGO/Cu(0.49), RGO/Cu(0.64), RGO/Cu(0.85) and RGO/Cu(1.31) are 467, 474, 553, 490, 498 and 454 MPa, respectively (Figure 6a). The tensile yield strength of RGO/Cu composites increases first and then decreases as the volume fractions of RGO increase. It can be inferred that the RGO is quite effective as a reinforcement in the Cu matrix; however, excessive graphene could agglomerate in the copper matrix, which reduces the properties of the composites. A typical characteristic of ductile fracture can be seen in the well-developed dimples and tear ridge structures on the fracture surfaces of RGO/Cu composites (Figure S4).

Generally, the mechanical properties of the RGO/Cu composites are determined by the unique characteristics of RGO, the homogeneous dispersion of the RGO and the interfacing bonding that occurs between the RGO and Cu matrix. The RGO/Cu (0.49) composite is the basis for this discussion.

(1) Dislocations strengthening mechanism

A high–density dislocation zone can be formed at the interface between the RGO and Cu matrix due to the different thermal expansion coefficients during the cooling of the SPS process. And the expression is as follows [30,31]:

(1) $$∆\sigma_{CET}=kG_{M}b\sqrt{12\frac{∆\alpha ∆TV_{G}}{bL_{G}}}$$

where *k* is a constant (1.25). The Cu matrix has a shear modulus called *G_M_* (42.1 GPa). The Burgers vector is represented by *b* (0.256 nm) in the matrix [32]. Δ*α* (Δ*α* = *α*_Cu matrix_ − *α*_RGO_, *α*_Cu matri*x*_ = 17.7 × 10^−6^ °C^−1^, *α*_RGO_ = −8 × 10^−6^ °C^−1^) is the difference between the CTE of the Cu matrix and RGO. The sintering and test temperature are compared by Δ*T*, Δ*T* = 675 °C, and *V_G_* is the volume fraction of RGO. *L_G_* (length of the RGO) calculated by the TEM image of the composite is 190.8 (Figure S5).

(2) Grain refinement mechanism

The Hall–Petch equation is used to analyze the grain refinement process of the RGO/Cu composite [33]:

(2) $$∆\sigma_{Hall-petch}=K\left(d_{c}^{-\frac{1}{2}}-d_{0}^{-\frac{1}{2}}\right)$$

where *d*_0_ and *d*_c_ are the grain size of RGO/Cu(0) and RGO/Cu composite, respectively, as shown in Figure 6a,c. *K* is the Hall–Petch slope (140 MPa μm^1/2^ for the Cu) [32].

(3) Load transfer strengthening mechanism

Load-transfer strengthening plays an important role in the enhanced strength of metal matrix composites [13,34]. The strong interfacial adhesion allows for the transfer of the load from the Cu matrix to the RGO. The modified shear-lag model can be used to calculate it, as follows [13]:

(3) $$∆\sigma_{yc}=\left(\frac{s+4}{4}V_{G}+V_{M}\right)\sigma_{ym}$$

where *V_G_* is the same as defined in Equation (1), *V_M_* is the volume fraction of the Cu matrix and *V_G_* + *V_M_* = 1. The yield strength of the pure Cu and RGO/Cu composite are *σ_ym_* and *σ_yc_*, respectively. The ratio of the side length (*L*_G_) to thickness (*t*) of RGO in the composite is *s*. The *s* calculated by the TEM image of the composite is 62.3 (Figure S5). The load transfer effect results in an increase in yield strength that can be expressed by the following:

(4) $$\sigma yc=\sigma ym+∆\sigma LT\sigma_{yc}=\sigma_{ym}+∆\sigma_{LT}$$

by combining Equations (3) and (4).

(5) $$∆\sigma_{LT}=\frac{s}{4}V_{G}\sigma_{ym}$$

(4) Orowan strengthening mechanism

The yield strength enhancement of the RGO/Cu composite can be calculated by the Orowan–Ashby equation [35]:

(6) $$∆\sigma_{Orowan}=\frac{0.13G_{M}b}{L_{G}\left[{\left(\frac{1}{2V_{G}}\right)}^{\frac{1}{3}}-1\right]}\ln\frac{L_{G}}{2b}$$

where *G_M_*, *b*, *V*_G_ and *L*_G_ are equivalent to defined in Equations (1) and (3).

Hence, the yield strength of the RGO/Cu composite can be determined by estimating the following [36]:

(7) $$\sigma_{yc}=\sigma_{ym}+\sqrt{∆{\sigma^{2}}_{CET}+∆{\sigma^{2}}_{Hall-petch}+∆{\sigma^{2}}_{LT}+∆{\sigma^{2}}_{Orowan}}$$

Figure 6b displays the theoretical calculation of the strengthening mechanisms, and this result reveals that the thermal mismatch mechanism, grain-size refinement and load transfer are the main strengthening mechanisms of the RGO/Cu composite.

It is possible that the homogenous dispersion of RGO in the Cu matrix is responsible for the strong effect of the thermal mismatch mechanism on strengthening calculations. Generally, the Orowan strengthening mechanism is important in nano-size-reinforced metal matrix composites. However, taking into account the volume fraction and size of graphene, the contribution of the Orowan strengthening mechanism of this study could only slightly strengthen the composites, which is consistent with some other research results [13,20].

Figure 6c illustrates the contrast between theoretical and experimental values, indicating that the tensile yield strength increase observed in the test is lower than that of the calculated value. The strengthening effect could be weakened by processing-induced defects in graphene, which is a possible reason. Another possible reason is that the enhancement effect is not accurately estimated by the calculated model because of a systematic error, such as the load transfer effect’s shear-lag calculation model assumes that all graphene sheets are alignment with the load direction (Equation (5)). The load transfer effect may be partially affected by the fact that not all graphene in the composite is aligned with the extrusion direction. Figure 6d shows the tensile stress–strain curves for the RGO/Cu-R composites. It can be found that the hot-rolling process significantly enhances the elongation of composites. Deeper and more uniform dimples are present on the fracture surfaces of the RGO/Cu-R composites (Figure S6) compared to the RGO/Cu composites (Figure S4). The indicator shows that the RGO/Cu-R composites possess excellent plasticity, which is in line with the outcomes of the tensile stress–strain test. As shown in Figure 6e, this work provides an exceptional balance of tensile strength and ductility, far superior to those of RGO/Cu copper matrix composites that have been reported earlier [17,18,19,20,21,22,23,37,38,39,40]. It can be easily found that the RGO/Cu(0.49)-R composite exhibits a well-balanced combination of tensile strength and ductility, which goes beyond the previously reported RGO/Cu composites with either low strength or low ductility (or both low). For industrial applications of Cu matrix composites, it is crucial to have excellent strength and ductility.

### 3.5. Electrical and Thermal Conductivity Properties of Composites

**Figure 7:**
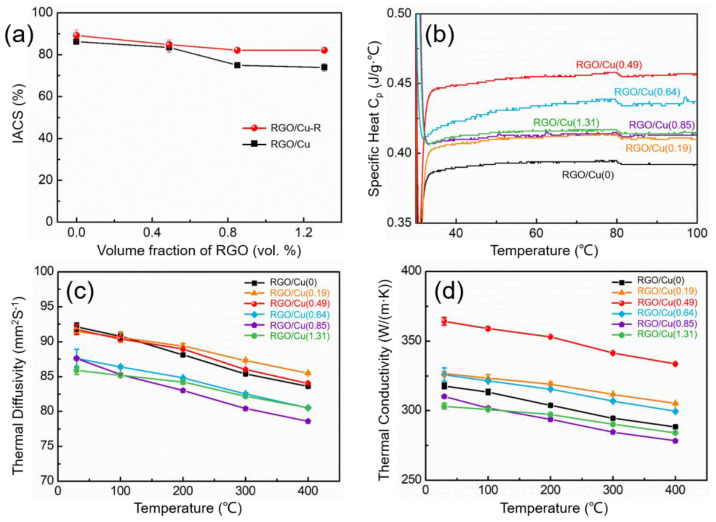
(**a**) The electrical conductivities of the RGO/Cu and RGO/Cu-R composites, (**b**) specific heat as a function of temperature, (**c**) thermal diffusivity and (**d**) thermal conductivity of Cu matrix and RGO/Cu composites.

Figure 7a shows the electrical conductivities of RGO/Cu and RGO/Cu-R composites as demonstrated by IACS. It can be found that as the volume fraction of RGO increases, the conductivity values of RGO/Cu composite slightly decrease. The hot-rolling process resulted in a significant improvement in the conductivity values of RGO/Cu composites. The reason may be that the RGO tends to align with the RD in the composites after the hot-rolling procedure.

The representative curve of specific heat (*C*_p_) for RGO/Cu composites is shown in Figure 7b. *C*_p_ increases first and then decreases with the increase in volume fractions of the RGO, and that of the RGO/Cu(0.49) composite is the highest. The average apparent thermal diffusivity (*α*) of the Cu matrix and RGO/Cu composite decreases a little with the increase in temperature, as shown in Figure 7c. The reason this is possible is that the thermal motion of the copper lattice is intensified with the increase in temperature, which hinders the motion of electrons in the heat transfer medium in the Cu matrix [33,34]. *α* is decreased gradually with the increase in volume fractions of RGO at 25 °C, and it may be that RGO between the Cu grain boundary impedes the movement of electrons and thus reduces the *α*. However, the thermal motion of the copper lattice in the RGO/Cu composites is limited by RGO with the increase in temperature, which reduces the hindrance to the motion of electrons; therefore, the *α* of RGO/Cu(0.19) and RGO/Cu(0.49) is slightly higher than the that of Cu matrix in the temperature range of 150 to 400 °C. The thermal conductivity (K) was calculated using the equation K = ρ*αC*_p_, where ρ is the density of the composites. Figure 7d shows the average thermal conductivity of the Cu matrix and RGO/Cu composites. The K is firstly increased and then decreases with the increase in volume fractions of the RGO at 25 °C, and that of the RGO/Cu(0.49) composite is up to 364 W/m·K, which is 14.5% higher than that of the Cu matrix.

## 4. Conclusions

The high strength, high electrical conductivity and high thermal conductivity RGO/Cu composites are fabricated by directly heating the GO/copper formate and SPS process. The results show that the oxygen-containing functional groups and defects of RGO are significantly reduced compared with those of GO. The microstructure analysis of the composites shows that RGO can be uniformly dispersed in the composites, and the average grain size of the composites decreases with the increase in the RGO volume fraction. If the volume fraction of RGO is above 0.85 percent, the dispersion of RGO in the composites is uneven, and the carbon-rich and carbon-poor regions appear. The grain size of Cu in the carbon-rich region is smaller than that in the carbon-poor region. The strengthening mechanism of the composites was clarified, and the theoretical values of the tensile strength properties of the composites were calculated according to the strengthening mechanism. The theoretical values and experimental values are in good agreement, as shown by the results. The ductility and electrical conductivity of as-rolled composites are greatly improved. The tensile strength and ductility of the RGO/Cu(0.49)-R composite are even higher than those of the RGO/Cu(0)-R composite without RGO. This work presents a highly efficient method for fabricating high-performance RGO-reinforced Cu composite for commercial application. The industrial production of this method is hindered by the obstacle posed by large-scale ball milling technology. In the future, our research will focus on further understanding the thermal conductivity mechanism, electrical conductivity mechanism and application of these composite materials in extreme environments.

## Figures and Tables

**Table 1:** The preparation of RGO/Cu composites necessitates the use of GO aqueous solution and copper formate tetrahydrate. The percentages related to weight of carbon for RGO/Cu composites measured using a high-frequency infrared carbon sulfur analyzer. The volume fractions of carbon and GO for RGO/Cu composites calculated by weight percentages of carbon.

Composites	GO Aqueous Solution (g)	Copper Formate Tetrahydrate (g)	Weight Percentages of Carbon (wt. %)	Volume fractions of Carbon (vol.%)	Volume Fractions of RGO (vol.%)
RGO/Cu(0)	0	142	0.026	0.10	0
RGO/Cu(0.19)	2.8	142	0.072	0.29	0.19
RGO/Cu(0.49)	5.6	142	0.148	0.59	0.49
RGO/Cu(0.64)	8.5	142	0.185	0.74	0.64
RGO/Cu(0.85)	11.4	142	0.237	0.95	0.85
RGO/Cu(1.31)	17.2	142	0.352	1.41	1.31

## Data Availability

The data underlying this article will be shared on reasonable request from the corresponding author.

## Supplementary Materials

The following supporting information can be downloaded at: https://www.mdpi.com/article/10.3390/ma17112519/s1, Figure S1: SEM image of GO. Figure S2: SEM images of (a) RGO/Cu(0) (b) RGO/Cu(0.19), (c) RGO/Cu(0.64), (d) RGO/Cu(0.85) and (e) RGO/Cu(1.31) composite powders. Figure S3: XRD patterns of (a) RGO/Cu(0) (b) RGO/Cu(0.19), (c) RGO/Cu(0.49), (d) RGO/Cu(0.64) (e) RGO/Cu(0.85) and (f) RGO/Cu(1.31) composites. Figure S4: SEM images of the fracture surfaces of (a) RGO/Cu(0), (b) RGO/Cu(0.19), (c) RGO/Cu(0.49), (d) RGO/Cu(0.64), (e) RGO/Cu(0.85) and (f) RGO/Cu(1.31) composites. Figure S5: (a) TEM images of RGO/Cu(0.49), the distribution of (b) length and (c) the ratio of the side length to thickness of the RGO. Figure S6: SEM images of the fracture surfaces of (a) RGO/Cu(0)-R, (b) RGO/Cu(0.49)-R, (c) RGO/Cu(0.85)-R and (f) RGO/Cu(1.31)-R composites.
