# Supplementary material for: Facile Preparation of High-Performance Reduced Graphene Oxide (RGO)/Copper (Cu) Composites Based on Pyrolysis of Copper Formate
Source: Materials (Basel). 2024 May 23;17(11):2519. doi: 10.3390/ma17112519 (PMC11174114; doi:10.3390/ma17112519)
Supplement: Supplementary file 1 [file materials-17-02519-s001.zip › materials-2992103-supplementary.pdf]

Supplementary File

Facile Preparation of High-performance Reduced Graphene Oxide (RGO)/Copper (Cu) Composites based on Pyrolysis of Copper Formate

Zhendong Shi[a,b*], Qingwen Yun [a], Tong Zhang[c], Changsheng Xing[c], Jie Li[c], Yunzhong Wu[c] and Lidong Wang[c, **]

[a]*Harbin Aircraft Industry Group Co.Ltd, Aviation Industry Corporation of China, Harbin 150066, PR China*

[b]*Harbin Hafei Aviation Industry Co.Ltd, Aviation Industry Corporation of China, Harbin 150010, PR China*

[c]*School of Materials Science and Engineering, Harbin Institute of Technology, Harbin, 150001, China.*

*Corresponding author. E-mail: hitszd@163.com (Z.D. Shi)

*Corresponding author. E-mail: wld@hit.edu.cn (L.D. Wang)

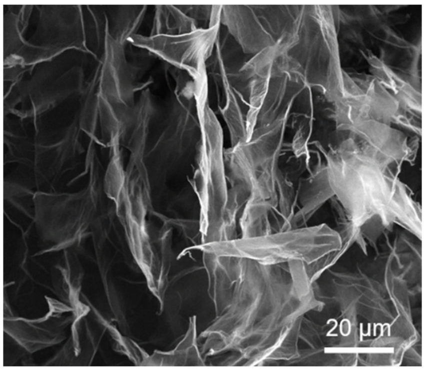

Fig. S1 SEM image of GO.

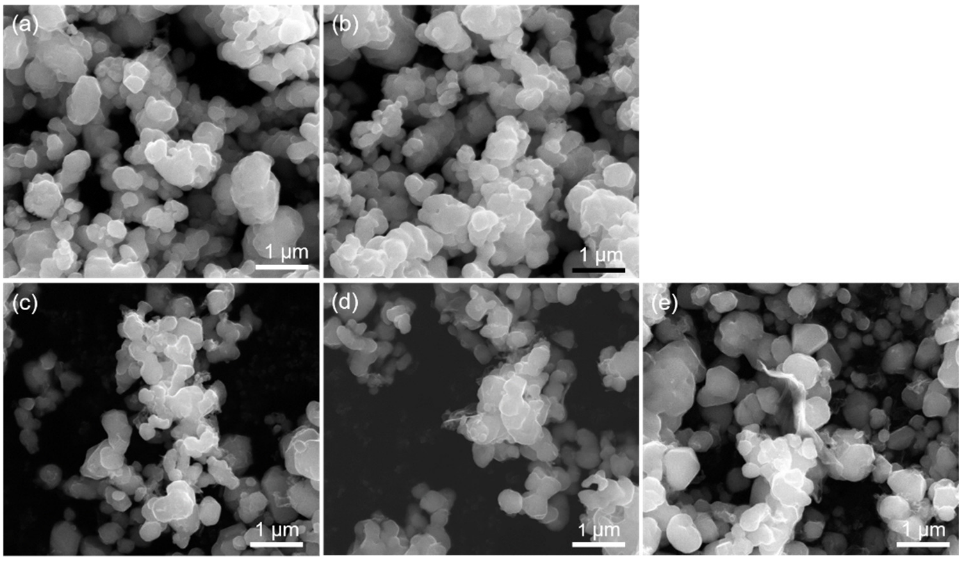

Figure S2. SEM images of (a) RGO/Cu(0) (b) RGO/Cu(0.19), (c) RGO/Cu(0.64), (d) RGO/Cu(0.85) and (e) RGO/Cu(1.31) composite powders.

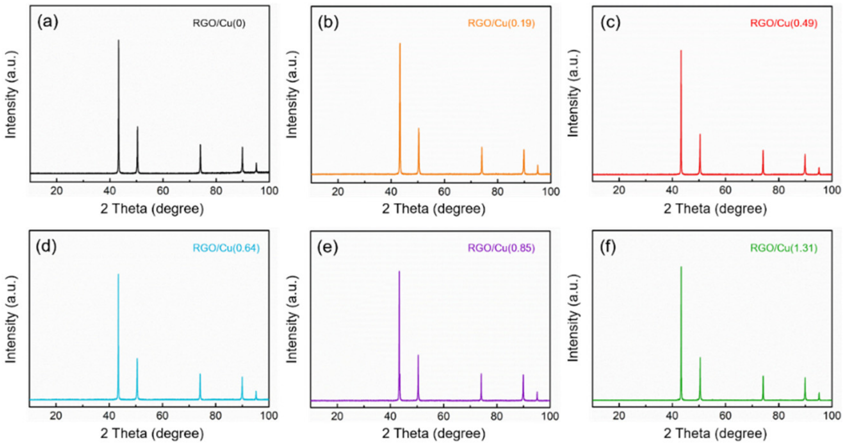

Figure S3. XRD patterns of (a) RGO/Cu(0) (b) RGO/Cu(0.19), (c) RGO/Cu(0.49), (d)

RGO/Cu(0.64) (e) RGO/Cu(0.85) and (f) RGO/Cu(1.31) composites.

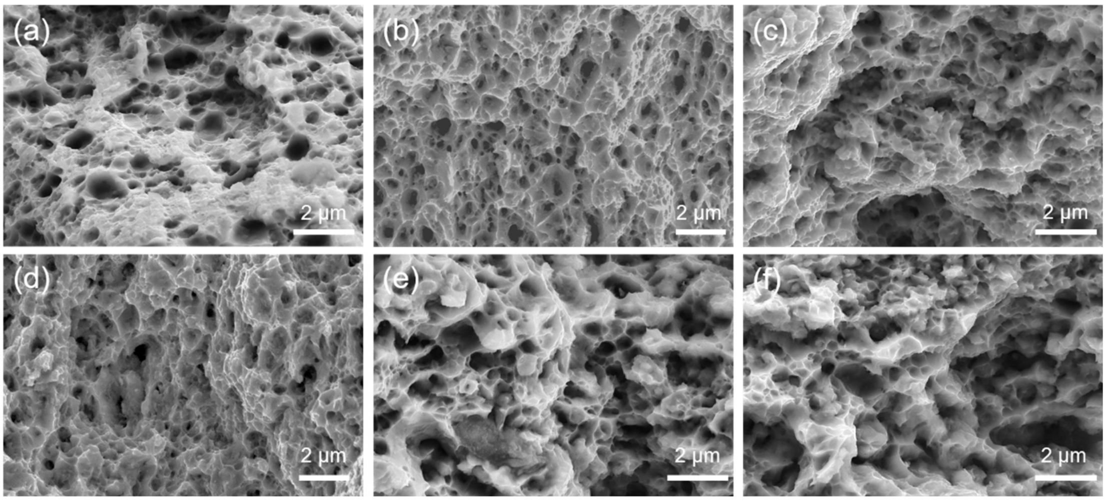

Figure S4. SEM images of the fracture surfaces of (a) RGO/Cu(0), (b) RGO/Cu(0.19), (c) RGO/Cu(0.49), (d) RGO/Cu(0.64), (e) RGO/Cu(0.85) and (f) RGO/Cu(1.31) composites.

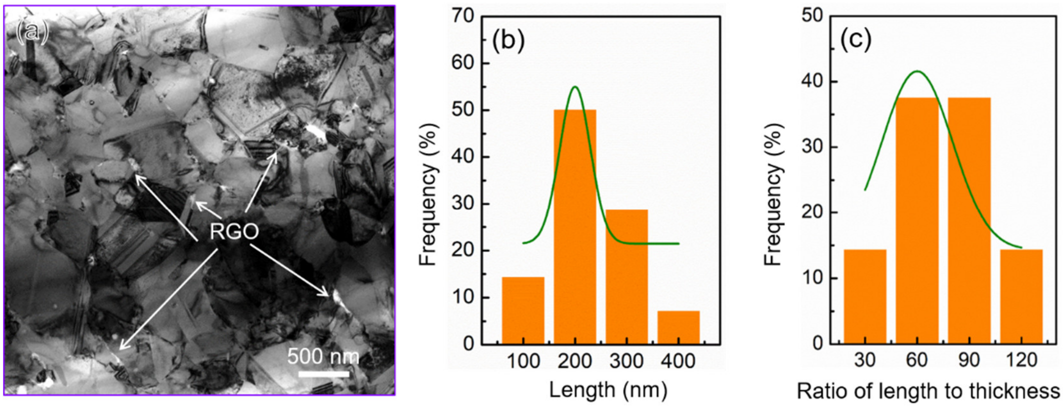

Figure S5. (a) TEM images of RGO/Cu(0.49), the distribution of (b) length and (c) the ratio of the side length to thickness of the RGO.

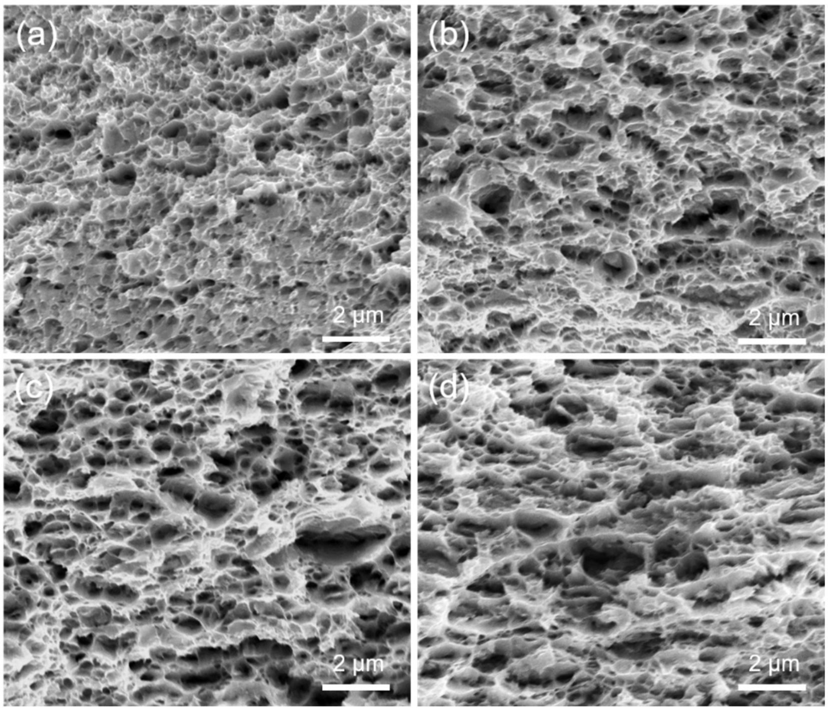

Figure S6. SEM images of the fracture surfaces of (a) RGO/Cu(0)-R, (b) RGO/Cu(0.49)-R, (c) RGO/Cu(0.85)-R and (f) RGO/Cu(1.31)-R composites.
